# Corporate capture of public health by food industries in Europe

**DOI:** 10.1093/eurpub/ckac129.091

**Published:** 2022-10-25

**Authors:** M Royo Bordonada

**Affiliations:** National School of Public Health, Institute of Health Carlos III, Madrid, Spain

## Abstract

Corporate capture is the deliberately planned process whereby political decisions respond to a particular interest of a private nature, in detriment to the public interest. The result is an unjust regulation or the absence of regulation where this is necessary for the protection of the common good. Corporate capture of public health has to do with commercial determinants of health, such as alcohol, tobacco, sugar-sweetened beverages and ultra-processed foods. Capture-related actions are targeted at civil society, experts, public-health officials, civil servants and politicians. These actions range from material (corruption, revolving doors and donations to political parties) to intellectual (distortion of science and professional training), social (control of information and communication) and/or cultural (group identity, status and relationship of the regulator with the representatives of private corporations). The most common capture strategies are aimed at biasing scientific results, creating consumers from an early age, promoting a good image of corporations, questioning the legitimacy and appropriateness of governmental intervention aimed at regulating their activity, controlling professional education; and exerting pressure on governments and international bodies. To illustrate this phenomenon, we present findings from several examples of corporate capture of food policies in Europe. Preliminary results from publicly available information suggest that most of the mentioned tactics were used in Europe in order to block nutritional profiles, health warnings on food and effective food advertising regulations. We suggest implications for how European legislation can better protect European citizens, especially children, from vested interests aimed at promoting the consumption of unhealthy food and beverages.

